# Low doses of ivermectin cause sensory and locomotor disorders in dung beetles

**DOI:** 10.1038/srep13912

**Published:** 2015-09-09

**Authors:** José R. Verdú, Vieyle Cortez, Antonio J. Ortiz, Estela González-Rodríguez, Juan Martinez-Pinna, Jean-Pierre Lumaret, Jorge M. Lobo, Catherine Numa, Francisco Sánchez-Piñero

**Affiliations:** 1 I.U.I. CIBIO, Universidad de Alicante, Alicante E-03080, Spain; 2 Departamento de Química Inorgánica y Química Orgánica. Universidad de Jaén, Campus Las Lagunillas, Jaén E-23071, Spain; 3 Departamento de Enfermería, Universidad de Alicante, Alicante E-03080, Spain; 4 Departamento de Fisiología, Genética y Microbiología. Universidad de Alicante, Alicante E-03080, Spain; 5 UMR 5175 CEFE, CNRS - Université de Montpellier - Université Paul-Valéry Montpellier – EPHE, Université Paul-Valéry Laboratoire Zoogéographie, route de Mende, 34199 Montpellier cedex 5, France; 6 Museo Nacional de Ciencias Naturales-CSIC, Departamento de Biogeografía y Cambio Global. José Abascal 2, Madrid E-28006, Spain; 7 IUCN-Centre for Mediterranean Cooperation. Marie Curie 22, Campanillas, Málaga E-29590, Spain; 8 Departamento de Zoología, Universidad de Granada, Granada E-18071, Spain

## Abstract

Ivermectin is a veterinary pharmaceutical generally used to control the ecto- and endoparasites of livestock, but its use has resulted in adverse effects on coprophilous insects, causing population decline and biodiversity loss. There is currently no information regarding the direct effects of ivermectin on dung beetle physiology and behaviour. Here, based on electroantennography and spontaneous muscle force tests, we show sub-lethal disorders caused by ivermectin in sensory and locomotor systems of *Scarabaeus cicatricosus*, a key dung beetle species in Mediterranean ecosystems. Our findings show that ivermectin decreases the olfactory and locomotor capacity of dung beetles, preventing them from performing basic biological activities. These effects are observed at concentrations lower than those usually measured in the dung of treated livestock. Taking into account that ivermectin acts on both glutamate-gated and GABA-gated chloride ion channels of nerve and muscle cells, we predict that ivermectin’s effects at the physiological level could influence many members of the dung pat community. The results indicate that the decline of dung beetle populations could be related to the harmful effects of chemical contamination in the dung.

Avermectins are macrocyclic fermentation products of *Streptomyces avermilitis*. Subsequent chemical modifications resulted in the synthesis of ivermectin (22,23-dihydroavermectin B_1_), which is marketed as a mixture containing at least 80% 22,23-dihydroavermectin B_1a_ and no more than 20% 22,23-dihydroavermectin B_1b_^1^,^2^. Ivermectin is generally used to control the ecto- and endoparasites (mites and nematodes, mainly) of livestock and pets (dogs and cats)^3^,^4^. Ivermectin is also used in antifilarial chemotherapy in humans as it kills the immature worms (microfilariae) that cause the harmful symptoms of onchocerciasis^5^,^6^.

The mode of action of ivermectin is very effective, being 25 times more potent than all currently available veterinary pharmaceuticals^6^. The main physiological response to ivermectin is an increase in plasma membrane permeability due to an agonistic action on chloride channels present in nerve and muscle cells^7^. This effect results in a decreased membrane input resistance and hence in a reduced probability of action potential generation. Ivermectin acts as a positive allosteric regulator of several ligand-gated channels including γ–aminobutyric acid (GABA)-gated chloride channels and glutamate-gated chloride channel (GluCl). As these channels are inhibitory, the effect of ivermectin is a potentiation of the inhibitory neurotransmission produced by GABA and glutamate in invertebrates, which culminates in a paralysis of somatic muscles^8^,^9^,^10^. GABA-gated chloride ion channels are present in neurons and abundant in local interneurons and antennal lobes of insects^11^,^12^,^13^,^14^ and are essential for olfactory processing. Evidence suggests that GABA released from local interneurons activates GABA receptors in the presynaptic axon terminals of olfactory sensorial neurons-axons, leading to presynaptic inhibition and thereby reducing synaptic transmission. Recently, GABA_B_ receptors have been observed in olfactory sensory neurons in the male antenna of *Heliothis virescens*, instantiating a GABA-mediated gain control mechanism that could play a pivotal role in processing pheromone signals^15^. Thus, due to its action on both GluCl and GABA ion channels, target organisms affected by ivermectin are generally ecto- and endoparasites of livestock, pets and humans; however, ivermectin sensitivity has been described as an ancient trait affecting potentially all Ecdysozoan (moulting animals) species^16^.

After ivermectin is administered to mammals, its chemical decomposition by metabolism is generally low^17^,^18^, and between 62% and 98% of the ivermectin inoculated may be excreted as unchanged residue in faeces^19^,^20^. Unfortunately, ivermectin excreted by cattle, sheep, goats, swine, horses, etc. seems to have strong non-target effects on beneficial arthropods such as the arthropod community responsible for decomposing livestock dung. It is commonly recognised that adverse effects on coprophilous insects such as flies and beetles occur as a consequence of highly toxic concentrations of ivermectin in the dung of treated livestock^19^,^21^. Among non-target organisms, dung beetles are the most important group in terms of diversity, abundance, biomass and functional relevance (or ecosystem services) within the residents of dung pat communities^22^. For this reason, several studies on the ecotoxicology of ivermectin published during the last decades have focused on this group^23^. It is assumed that larvae of dung beetles are more sensitive to ivermectin residue (and that of other endectocides) than mature dung beetles^21^. In fact, some veterinary manuals indicate that mature dung beetles are usually unaffected by macrocyclic lactone residues found in dung^23^. However, only scarce data exist about the direct effects of ivermectin on mature dung beetles. Currently, the published data refer to a few mortality cases of newly emerged adults and alteration of oviposition processes^24^,^25^,^26^,^27^,^28^. Many experimental studies have focused on breeding behaviour and offspring survival, but no data are available on the direct effects of ivermectin on the physiology and health status of mature dung beetles.

In this study, the model species was *Scarabaeus cicatricosus* Lucas, which was selected due to its local abundance and functional relevance in Mediterranean ecosystems. The genus *Scarabaeus* (Coleoptera, Scarabaeinae) includes 30 recognised species in the Palaearctic region^29^, all being large rollers that roll balls made of excrement far from the dung source for feeding or nesting. Studying the historical occurrence of the seven *Scarabaeus* species inhabiting the Iberian Peninsula, a previous investigation showed that these species have suffered a general decline^30^, also apparent in other European regions^31^,^32^,^33^. *Scarabaeus cicatricosus* is an endemic species mainly restricted to the Atlantic coast and sandy soils of the southwestern Iberian Peninsula, its populations being well established only within the Doñana Biological Reserve (DBR-ICTS), an ivermectin-free site within the Doñana National Park (Huelva) in southern Spain^34^,^35^. Taking into account that the use of ivermectin has been prohibited in this reserve and that the high abundance and biomass of this beetle species (approximately 1.5 grams of fresh weight) make it a keystone species from a functional perspective, we decided to use ‘healthy’ specimens of this species collected from uncontaminated localities to examine the effects of ingesting ivermectin. Thus, the existence of harmful effects of ivermectin on this species could support a probable role played by chemical contaminants in the decline of dung beetles.

We explored the sub-lethal symptoms and lethal consequences of ivermectin treatment in adults of this key dung beetle species using two different physiological methods combined with behavioural assays. Because ivermectin effects binding sites in GABA-gated chloride channels found in nervous system and olfactory sensory neurons (OSN) of insects, the first objective was to analyse the effect of this substance on the sensorial response of antennae using electroantennography (EAG). EAG is a method widely used in entomological research for the detection of volatiles perceived by the antennal olfactory apparatus of insects. In dung beetles, antennal clubs have the function of detecting food resources and close-range detection of sexual or aggregation signals^36^. If ivermectin affects OSN in dung beetles, we hypothesised that a decrease of antennal EAG responses would have to take place. Secondly, given that ivermectin acts on both glutamate-gated chloride ion channels and GABA-gated chloride ion channels in nerve and muscle cells generating paralysis of somatic muscles, we also aimed to examine the effect of ivermectin on the power output of synchronous walking leg muscles of dung beetles. In nematodes, exogenous glutamate inhibits pharyngeal pumping, which is mimicked by ivermectin^37^, whereas paralysis of somatic muscles is associated with GABA-gated chloride channel receptors^38^. Ivermectin inhibits pharyngeal muscle contractions^39^,^40^, and in insects such as mosquitoes, ivermectin causes paralysis and mortality in adults^41^,^42^,^43^. Thus, we hypothesised that the ingestion of ivermectin by mature dung beetles could induce a decrease of muscle force contraction, possible ataxia and death. Finally, to complement the formerly mentioned physiological information, we carried out a complementary behavioural study using an olfactometer bioassay, which combines testing both olfactory and locomotor (walking) capacities.

## Results

### Effect of ivermectin on the antennal olfactory apparatus

**Figure 1:**
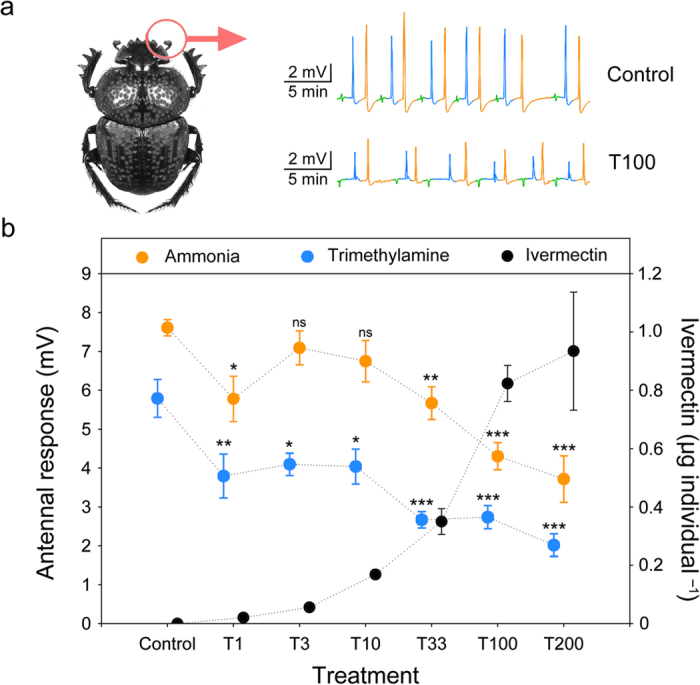
Ivermectin decreases the olfactory response of *Scarabaeus cicatricosus*. (**a**) Electroan-tennography recordings of an ivermectin-free individual (control) and an individual treated with application of ivermectin in dung (100 μg kg^–1^ dung (fresh weight)) after exposure to hexane as a standard reference compound (green line) and two model odorants: ammonia (orange line) and trimethylamine (blue line). (**b**) Mean (±s.e.m.) data showing the effect of different ivermectin doses on sensitivity to ammonia (orange dots) and trimethylamine (blue dots). Six concentrations of 1.0, 3.3, 10.0, 33.3, 100.0, and 200.0 μg kg^–1^ dung (fresh weight) plus an untreated control were used. Black dots correspond to ivermectin ingested for each treatment (*P < 0.05, **P < 0.01, ***P < 0.001, post-hoc Dunnet’s test). Photo credit: José R. Verdú.

Electroantennography measurements showed that ivermectin ingestion negatively affected the antennal olfactory apparatus of *S. cicatricosus* (Fig. 1 and supplementary Table S1). The concentration of ivermectin in the dung treatments was positively related to the quantity of ivermectin ingested per individual (*R*^*2*^ = 0.63; df = 1.13; *P* < 0.0001; *n* = 10), showing that the addition of different doses of ivermectin to the dung placed in the container effectively increased the concentration of ivermectin in the beetles. Statistically significant differences were found between the experimental treatments (*F* = 9.26; df = 6; *P* < 0.0001; *n* = 10), but neither body mass nor sex had a significant effect (*P* = 0.71 and *P* = 0.68, respectively; *n* = 10 in both cases). Post-hoc Dunnet’s tests indicated statistically significant differences between the control and all ivermectin treatments being more significant from doses above 33.3 μg kg^–1^ dung (fresh weight) (Fig. 1). This pattern was repeated when the ammonia odorant was used. Statistically significant differences between the experimental groups were also obtained (*F* = 8.61; df = 6; *P* < 0.0001; *n* = 10), and neither body mass nor sex was significant (*P* = 0.19 and *P* = 0.76, respectively; *n* = 10 in both cases). Post-hoc Dunnet’s test showed significant differences between the control and strongest ivermectin treatments being more notable from doses of 100.0 μg kg^–1^ dung (fresh weight) (Fig. 1).

### Effect of ivermectin on spontaneous muscle force

**Figure 2:**
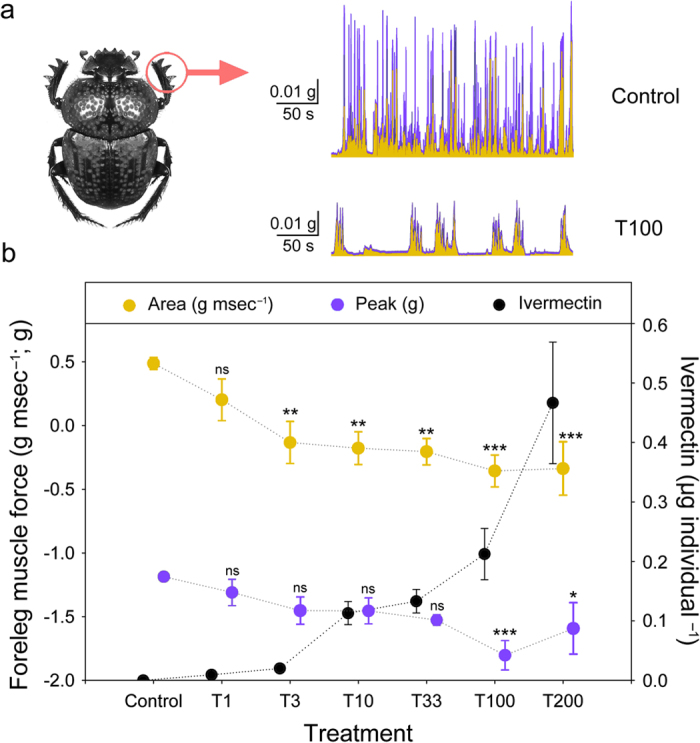
Ivermectin decreases the muscle force of *Scarabaeus cicatricosus*. (**a**) Spontaneous isometric muscle contraction recordings of an ivermectin-free individual (control) and an individual treated with application of ivermectin in dung (100 μg kg^–1^ dung (fresh weight)). (**b**) Mean ( ±  s.e.m.) data showing the effect of different ivermectin doses on foreleg muscle force. The treatments correspond to 1.0, 3.3, 10.0, 33.3, 100.0, and 200.0 μg kg^–1^ dung (fresh weight) plus an untreated control. Yellow dots correspond to the area of spontaneous force generation above the resting level during a time interval of 5 minutes (in g ms^–1^); purple dots correspond to the peak (maximum value of spontaneous force; in g) during a time interval of 5 minutes (data are log10 transformed means ± s.e.m.; see text for statistical results); black dots correspond to the ivermectin ingested for each treatment (data are means ± s.e.m.; *P < 0.05, **P < 0.01, ***P < 0.001, post-hoc Dunnet’s test). Photo credit: José R. Verdú.

Spontaneous isometric force decreased with ivermectin ingestion (Fig. 2 and supplementary Table S1). A clear relationship between ivermectin concentration in the treatment and the quantity of ivermectin ingested per individual was observed (*R*^*2*^ = 0.66; df = 1.52; *P* < 0.0001; *n* = 10). For SMF for the ‘Area’ variable (see methods section), significant differences between the experimental groups were obtained (LogArea: *F* = 4.80; df = 6; *P* < 0.0001; *n* = 10), whereas body mass and sex were not significant (*P* = 0.41 and *P* = 0.97, respectively; *n* = 10 in both cases). Post-hoc Dunnet’s tests showed significant differences between the control and strongest ivermectin treatments being more significant from doses of 100.0 μg kg^–1^ dung (fresh weight) (Fig. 2). This result was similar to those obtained for the ‘Peak’ variable showing significant differences between the experimental groups (LogPeak: *F* = 3.65; df = 6; *P* < 0.001; *n* = 10), whereas body mass and sex were not significant (*P* = 0.41 and *P* = 0.97, respectively; *n* = 10 in both cases). Post-hoc Dunnet’s tests showed significant differences between the control and both T100 and T200 (Fig. 2). The fact that both the average muscle force and the peak muscle force were affected suggests that ivermectin could alter the locomotory behaviour of beetles in the field.

### Effect of ivermectin on foraging behaviour

**Figure 3:**
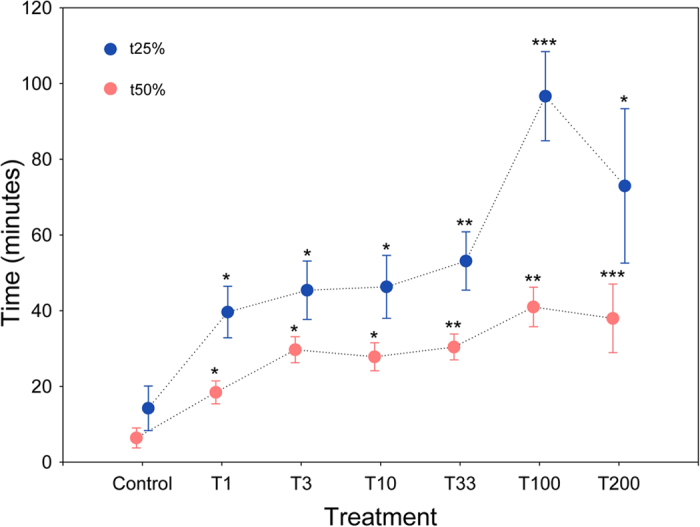
Ivermectin alters the foraging behaviour of *Scarabaeus cicatricosus*. Mean (±s.e.m.) data showing the negative effects of different ivermectin doses on the time required to detect and arrive at a food resource. Six concentrations of 1.0, 3.3, 10.0, 33.3, 100.0, and 200.0 μg kg^–1^ dung (fresh weight) plus an untreated control were used. Red dots indicate the time required for 25% of individuals to arrive (t25%; 5 individuals); blue dots correspond to the time required for 50% of individuals to arrive (t50%; 10 individuals) (data are means ± s.e.m.; *P < 0.05, **P < 0.01, ***P < 0.001, post-hoc Dunnet’s test).

Olfactometer tests showed a significant negative effect of ivermectin on foraging success (Fig. 3 and supplementary Table S1). The time elapsed to detect and to arrive at a food source increased significantly when the concentration of ivermectin was greater (*F*_t25%_ = 9.64, df = 6, *P* < 0.0001, *n* = 10; *F*_t50%_ = 8.11, df = 6, *P* < 0.0001, *n* = 10). t25% in post-hoc Dunnet’s tests showed significant differences between the control and strongest ivermectin treatments (Fig. 3). In the case of t50%, post-hoc Dunnet’s tests showed significant differences between the control and all ivermectin treatments (Fig. 3).

### Lethal effects of ivermectin

**Figure 4:**
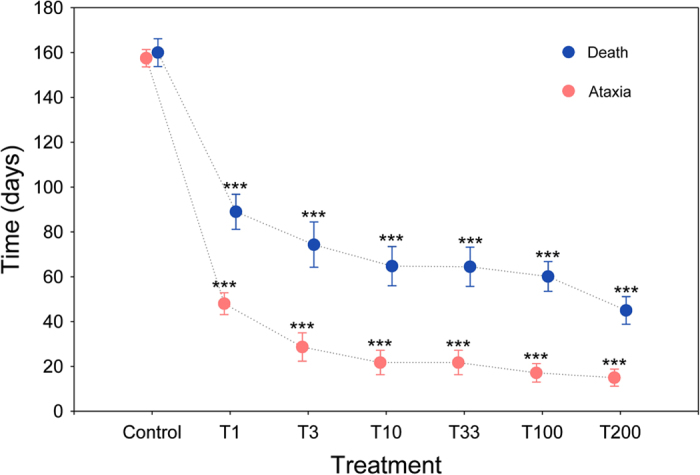
Ivermectin causes paralysis and death of *Scarabaeus cicatricosus.* Mean (±s.e.m.) data showing the effect of different ivermectin doses on ataxia and subsequent death of individuals. Six concentrations of 1.0, 3.3, 10.0, 33.3, 100.0, and 200.0 μg kg^–1^ dung (fresh weight) plus an untreated control were used. Red dots correspond to the time required to cause ataxia, whereas blue dots indicate the time at which death occurred (data are means ± s.e.m.; *P < 0.05, **P < 0.01, ***P < 0.001, post-hoc Dunnet’s test).

Ingestion of ivermectin brought about a reduction in mobility, ataxia-paralysis and finally death in the adults of *S. cicatricosus* (Fig. 4 and supplementary Table S1). The time required to arrive at irreversible effects decreased as the dose of ivermectin increased (*F*_Ataxia_ = 139.4, df = 6, *P* < 0.0001, *n* = 20; *F*_Death_ = 36.7, df = 6, *P* < 0.0001, *n* = 20). In both cases, post-hoc Dunnet’s tests showed significant differences between the control and all ivermectin treatments (Fig. 4).

## Discussion

The sub-lethal and lethal effects of ivermectin residue on mature dung beetles were studied in terms of their physiology and behaviour. Ivermectin affects the antennal olfactory apparatus in *S. cicatricosus* adults, decreasing their sensorial capacity markedly as the amount of ivermectin ingested increases. This result seems to be functionally related to the existence of ivermectin binding sites in GABA-gated chloride channels observed both in insect nervous system and olfactory sensory neurons^15^. Evidence about the presence of GABA-gated chloride ion channels in olfactory and mechanosensory organs in arthopods^15^,^44^,^45^ offers a new line of research exploring the mode of action and toxicology of ivermectin at the sensorial level. Our results show that the ingestion of very small doses of ivermectin is enough to considerably diminish the sensory capacity of individuals. The effective dose is appreciably lower than those usually measured in the dung of treated livestock. The concentration of ivermectin residue found in excreted dung varies according to the supply method, dosage and diet. For example, a reference study^46^ found ivermectin concentrations of 117.8–243.8 μg kg^–1^ dung (fresh weight) in fresh dung collected from animals 3 days after drug administration (500 μg kg^–1^ bw, topical application), and 42.6–61.6 μg kg^–1^ dung (fw) was found after 12 days. In a recent study^47^, the measured ivermectin concentration was 2.845 and 2.480 mg kg^–1^ in dung (dry weight) (~355.6 and 310.0 μg kg^–1^ dung fresh weight) collected from animals 3 and 7 days, respectively, after drug administration (500 μg kg^–1^ bw, topical application). After two weeks, the ivermectin concentration was still relatively high; 0.7 mg kg^–1^ (dry weight) (~ 86.5 μg kg^–1^ fresh weight). Even after 28 days, ivermectin was still detectable with 49.0 μg kg^–1^ (dry weight) (~ 6.1 μg kg^–1^ fresh weight). In our study, the lowest concentration of ivermectin used for treatment was notably lower (1.0 μg kg^–1^ fresh weight equivalent to 0.022 ± 0.001 μg individual^–1^ in our study) but still capable of adversely affecting the antennal olfactory apparatus. At higher concentrations (from 33.3 μg kg^–1^ of fresh weight or 0.347 ± 0.029 μg individual^–1^), we obtained stronger adverse effects even though these concentrations are markedly lower than those observed in the dung excreted by treated livestock. Thus, we provide the first evidence that environmentally realistic doses of ivermectin notably decrease the olfactory capacity of dung beetles, suggesting negative consequences on any activity that this sensorial apparatus facilitates, such as searching for food or a mate.

Furthermore, taking into account that ivermectin acts on both glutamate-gated chloride and GABA-gated chloride ion channels of nerve and muscle cells, inhibiting neurotransmission and resulting in paralysis of somatic muscles^48^,^49^, we also demonstrated the effect of ivermectin on neuromuscular activity. Our studies of spontaneous isometric contractions of foreleg muscles show that ivermectin affected the muscle force in adults of *S. cicatricosus*, considerably reducing the magnitude of spontaneous isometric contractions as the quantities of ivermectin ingested increased. It has been reported that the negative effects of ivermectin on muscle contractions is due to its action on glutamate and GABA ion channels, both in nematodes such as *Caenorhabditis elegans*, *Ascaris suum* and *Ascaridia galli*^40^,^50^,^51^ and in some insects such as *Drosophila melanogaster*^49^, *Schistocerca gregaria*^50^ and *Schistocerca americana*^52^.

In the present study, negative effects of ivermectin on spontaneous isometric contractions of foreleg muscles were observed from 3.33 μg kg^–1^ (fresh weight; equivalent to 0.020 ± 0.004 μg individual^–1^), similarly to the olfactory response, and again at a lower level than concentrations previously observed in dung excreted by treated livestock. Furthermore, behavioural assays showed a strong negative effect of ivermectin on foraging behaviour with a clear decrease in the capacity to detect a food source and arrive at it (Fig. 3). As in other insect species such as *D. melanogaster*, ivermectin causes an acute decrease of mobility^49^.

Sub-lethal effects such as those observed in this study imply that mature beetles feeding on dung, even at low concentrations of ivermectin, experience an acute toxicity that prevents the performance of normal biological activities such as food detection, intraspecific communication, locomotion and interaction with the environment. There are available data on the lethal effects of ivermectin on other mature dung beetles. Ivermectin can cause mortality of adults in some species such as *Copris ochus* and *Copris acutidens*^28^. Other studies^24^,^26^ have demonstrated that ivermectin delays maturation and increases the mortality of newly emerged adults fed with dung from animals 2–16 days after treatment. However, information about the non-lethal but harmful effects of ivermectin on dung beetles is lacking. In the present contribution it has been shown that survival time decreases drastically as the amount of ingested ivermectin increases, and pre-lethal symptoms such as ataxia and paralysis also exist (Fig. 4). Unfortunately, many studies have focused on the effects of ivermectin on larval stages and breeding behaviour^24^,^25^,^26^,^28^,^53^,^54^,^55^,^56^,^57^ without carrying out standardised studies directed at mature stages. In some cases, mortality was not observed in adults, such as in *Aphodius ater*, *Aphodius rufipes*^57^, and *Bubas bubalus*^24^, whereas mortality was observed in newly emerged beetles in *Copris hispanus*, *Onitis belial, Euoniticellus fulvus,* and *Onthophagus taurus*^24^,^26^. Differences between larval and adult stages could be due to differences in feeding behaviour^58^, whereas differences among species regarding ivermectin sensitivity could be due to diverse ecological and phylogenetic factors that occur with dung-dwelling sepsid flies^16^. Given that all Ecdysozoa are potentially susceptible to ivermectin toxicity, that there are phylogenetic signals associated with ivermectin sensitivity, and that even closely related species may have very different natural sensitivities to ivermectin^16^, we suggest the necessity of further studies able to assess ivermectin sensitivity in different dung beetles species from a phylogenetic point of view considering a combination of physiological (sensorial and mechanical capacities) and behavioural analyses using mature beetles.

In conclusion, the results obtained here demonstrate that adult dung beetles are susceptible to ivermectin toxicity with detectable sub-lethal effects in physiological and behavioural tests. This approach shows that ivermectin markedly alters the sensorial and locomotor capacity of adult dung beetles, preventing them from developing the most basic biological activities. In the case of this roller dung beetle species, the results suggest that the decline of these populations experienced across Europe might be related to the harmful effects of chemical contamination in dung due to the effects of avermectins. However, other veterinary medical products as organophosphates, which were commonly used in Europe during previous years may also contribute to the decline of *Scarabaeus* species^59^. From a practice point of view, we suggest the implementation of physiological testing in further eco-toxicological laboratory standardised tests^58^ to complement typical mortality tests (LC_50_ tests). Guidelines established by the International Cooperation on Harmonisation of Technical Requirements for Registration of Veterinary Medicinal Products (VICH; http://www.vichsec.org/) require an environmental risk assessment when animal excreted residues such as ivermectin are considered that adversely affect non-target organisms^21^. Therefore, we suggest that standardised tests are required, which incorporate more accurate analyses to test sub-lethal effects, using adult dung beetles to assess the sensorial and mechanical symptoms of novel veterinary pharmaceutical treatments.

## Methods

### Collection, selection and preparation of beetles

Individuals of *S. cicatricosus* were collected from the Doñana Biological Reserve (DBR-ICTS), an ivermectin-free site within the Doñana National Park (Huelva), in southern Spain during the summer (July 2013). All the individuals were maintained in plastic containers (60 × 40 × 40 cm) at 20 °C until their arrival at the laboratory, where they were sustained in a climate chamber at 29 ± 1: 21 ± 1 °C (L: D), 80 ± 5 RH with a photoperiod of 14:10 (L : D). These conditions mimic optimal conditions experienced in the field^34^.

To assure a common physiological state for all of the individuals, thus allowing the comparison of physiological and behavioural measures among the treatments, we selected the following: (a) only mature specimens according to external age-grading methods (e.g., tibia and clypeus erosion) that permit the identification of individuals of approximately the same age^60^; (b) mature individuals of the same body mass (approximately 1.2 g); (c) a sex ratio of 1:1 in each experiment. This work conforms to the Spanish legal requirements including those relating to conservation and welfare. Additionally, beetle collection was conducted with relevant permissions related to collection and field study in the Doñana National Park.

### Collection and preparation of dung

Non-contaminated bovine dung was obtained from ivermectin-free cattle in the Doñana Biological Reserve. Fresh dung was collected during the first two hours of the morning to avoid dung fauna colonisation as well as to minimise physical-chemical changes in the dung. If not used immediately, dung was stored frozen (−20 °C) or cold (3 °C) until its usage.

All the collected dung (~20 kg) was homogenised using an electric paint mixer. Ivermectin concentrations were selected according to literature and OECD recommendations^54^. Six concentrations of fresh dung, 1.0, 3.3, 10.0, 33.3, 100.0, and 200.0 μg kg ^–1^ plus an untreated control were used. Ivermectin solutions were made by dissolving ivermectin (Sigma-Aldrich Co.) in absolute ethanol (Sigma-Aldrich Co.), and 2 ml aliquots of the six selected concentrations were added to 2 kg portions of fresh dung, mixing all for 20 min by means of a kitchen machine mixer. For the untreated control, absolute ethanol (2 ml) was applied to the same quantity of dung. Residual ethanol was removed by evaporation during the 6 hours before transferring the dung treatments to individual experimental units.

### Ivermectin dosing

For electroantennography (EAG), foreleg muscle force and ataxia-mortality tests, each individual experimental unit consisted of a 15 × 10 × 7 cm plastic container using vermiculite as substrate. Dung treatments were supplied in 4 ml portions to better quantify the amount of ivermectin ingested per individual. Every three days, the dung not consumed was removed and measured (in ml), adding a new portion of the corresponding dung treatment. For EAG tests, beetles of each treatment were fed with treated dung for the 12 days before the bioassay was conducted. In the case of foreleg muscle force tests, the time of intoxication with ivermectin before bioassay was 12–18 days. For EAG and foreleg muscle force tests each treatment was replicated ten times and for ataxia-mortality tests each treatment was replicated twenty times. Beetles were numbered and weighed (body mass) prior to each treatment. In the case of olfactometer tests, beetles were also maintained in plastic containers (60 × 40 × 40 cm) with moist sterile vermiculite as substrate. Prior to testing, the beetles were supplied with dung during two weeks, replacing the dung every three days to ensure that the food supply was not a limiting factor. For this bioassay each treatment was replicated ten times.

### Electroantennogram recordings

Electroantennogram signals were recorded with an EAG system (Syntech, Kirchzarten, Germany) consisting of a universal single ended probe (Type PRS-1), a stimulus controller (CS-55), a data acquisition interface board (Type IDAC-02), and a stimulus air controller (Type CS-01). The antennae of *S. cicatricosus* were excised, inserted into small droplets of electrode gel (Spectra 360, Parker Laboratories, Fairfield, NJ, USA) and mounted individually between the electrodes in an antenna holder under a purified air flow (500 ml/min) (Supplementary Figure S1). A Syntech PC-based signal processing system was used to amplify and process the EAG signals. The signals were further analysed using the EAG 2000 software (Syntech, Kirchzarten, Germany).

Stimulation tests were carried out by applying puffs of humidified air (200 ml/min) flowing for 2 s using a stimulus controller through a Pasteur pipette containing a small piece of filter paper (Whatman no. 1) strip (1 cm^2^) with 1 μl of one of the test compounds flowing in a stainless steel delivery tube (1 cm diameter) with the outlet positioned at approximately 1 cm from the antenna. In each experiment, the antenna was first presented with an injection of the standard reference compound, hexane (HPLC grade, Sigma-Aldrich Co.), and then with injections of test odorants. Based on highly specific sensitivity of insect olfactory sensory neuron (OSNs) to several compounds related to decomposition of protein-containing organic materials, such as ammonia and amines^61^, standards of ammonia (25% v/v aqueous solution, Merck KGaA, Darmstadt, Germany) and trimethylamine (45% v/v aqueous solution, Sigma-Aldrich Co.) were selected as test odorants. Puffs of the tested compounds were applied at 1 min intervals at least 10 times on each antenna. Replicates were performed with different individuals (*n* = 10 for each treatment).

### Measuring spontaneous muscle force

The beetle was firstly immobilised using a bandage of polytetrafluoroethylene (PTFE) around the thorax and abdomen. After cutting off one of the anterior forelegs to avoid undesired movements, the beetle was set on a Sylgard base chamber in a sideways position using insect pins piercing the PTFE around its body. The remaining foreleg of the beetle was attached to a force transducer by a surgical thread tied between the femur and the tibia (the knot being cemented with cyanoacrylate adhesive) (Supplementary Figure S2). When the beetle was properly positioned, the femur was vertical with its major axis perpendicular to the force transducer (FORT 250, WPI Inc. Stevenage, UK). The force transducer was mounted in a micromanipulator, which allowed stretching of the leg until it was just taut. Spontaneous muscle force (SMF) was measured, recording for each individual isometric contractions during a period of 5 minutes. At the beginning of this time the leg was stretched to a position that evoked a maximal contraction. LabChart v.7 software (ADInstruments Inc., Dunedin, New Zealand) running in a PowerLab 8/35 (ADInstruments Inc. Dunedin, New Zealand) was used for data acquisition. The maximum spontaneous force (‘Peak’ variable; in g) and the integral of area of the spontaneous force in time (‘Area’ variable; in g ms) were measured using Microcal Origin (Microcal Software Inc., Northampton, MA, USA). Replicates were performed using different individuals (*n* = 10 for each treatment).

### Olfactometer test

Four four-arm olfactometers were used to test the possible effects of ivermectin on the olfactory detection of dung and on locomotion capacity. The olfactometers consisted of a central arena with sterile dry vermiculite as a substrate and four 5-cm diameter holes to attach tubes (arms) containing plastic containers with the test samples at the ends (Supplementary Figure S3). These olfactometers were developed and described in detail by Verdú *et al.*^62^. In this study, the plastic containers were designed to permit the entrance and exit of beetles that responded to the tested resources. In each olfactometer, two plastic containers were supplied with 20 g of fresh cattle dung and two empty containers were used as controls. Food sources were placed randomly in the olfactometer in each trial. The temperature in the bioassay room was maintained at 27–28 °C.

For each trial (*n* = 10 for each treatment), we counted the elapsed time that the beetles (20 beetles of each trial) took to go from the arena to the containers, measuring the time necessary to obtain both 25% (t25%; 5 individuals) and 50% (t50%; 10 individuals).

### Ataxia and mortality test

For each individual experimental unit described above, we supplied 4 ml of dung (either control or treated dung). Every three days we tested possible negative symptoms related to ivermectin ingestion performing two observations: a) coordinated walking, and b) reflex avoidance movements of the scape-pedicel joint of the antennae. When both observations were negative, we concluded that the beetle was healthy. Generally, both observations are sequential in time, thus if partial paralysis (ataxia) was observed in the legs and or antennae, we noted the date of observation and described the symptoms. In this assay, each treatment was replicated twenty times (*n* = 20).

### Statistical analyses

The data were analysed by an ANOVA design using General Linear Models (GLM) after log transformation of the dependent variables, if necessary, to follow a normal distribution. Normality was examined using a Kolmogorov-Smirnov test (P > 0.05 in all cases). Although the selection of beetles was made in order to reduce the variability of the body mass of individuals, we suspect that some differences in body mass among individuals may contribute to the random variability in the physiological measurements. Thus, we included body mass as a covariate in the analyses to reduce the error variance significantly. Multiple post-hoc comparisons between treatment groups against the control group were made using Dunnet’s tests (EAG test: mean of treatment < mean of control; SMF test: mean of treatment > mean of control; Olfactometer test: mean of treatment > mean of control; Ataxia and mortality test: mean of treatment > mean of control). The software STATISTICA v8.0 (StatSoft Inc, Tulsa, Oklahoma, USA) was used for all statistical analyses.

## Additional Information

**How to cite this article**: Verdú, J. R. *et al.* Low doses of ivermectin cause sensory and locomotor disorders in dung beetles. *Sci. Rep.* **5**, 13912; doi: 10.1038/srep13912 (2015).

## Supplementary Material

Supplementary Information
