# Supplementary material for: Low doses of ivermectin cause sensory and locomotor disorders in dung beetles
Source: Sci Rep. 2015 Sep 9;5:13912. doi: 10.1038/srep13912 (PMC4563563; doi:10.1038/srep13912)
Supplement: Supplementary Information [file srep13912-s1.pdf]

Supplementary information of the paper titled “Low doses of ivermectin cause sensory and locomotor disorders in dung beetles” by José R. Verdú, Vieyle Cortez, Antonio J. Ortiz, Estela González-Rodríguez, Juan Martinez-Pinna, Jean-Pierre Lumaret, Jorge M. Lobo, Catherine Numa and Francisco Sánchez-Piñero

Table S1. Mean values (±sem) of electroantennogram recordings (EAG) and spontaneous muscle force (SMF), and lethal effect tests.

Figure S1. Experimental setup for electroantennogram recordings.

Figure S2. Experimental setup for measuring spontaneous muscle force.

Figure S3. Experimental setup for olfactometer test.

Table S1. Mean values (±sem) of electroantennogram recordings (EAG), spontaneous muscle force (SMF), and lethal effect tests.

	EAG test (mV)		SMF test		Lethal effects (days)	
	Trimethylamine	Ammonia	‘Area’ (g ms^{-1})	‘Peak’ (g)	Ataxia	Death
Control	5.8 ± 0.5	7.6 ± 0.2	3.2 ± 0.4	0.07 ± 0.004	157.5 ± 7.0	160.0 ± 7.0
T1	3.8 ± 0.6	5.8 ± 0.6	2.3 ± 0.6	0.06 ± 0.01	48.0 ± 5.6	89.0 ± 13.5
T3	4.1 ± 0.3	7.1 ± 0.4	1.2 ± 0.4	0.04 ± 0.01	31.5 ± 3.1	76.4 ± 5.8
T10	4.0 ± 0.4	6.7 ± 0.5	0.9 ± 0.2	0.04 ± 0.01	21.8 ± 1.5	74.8 ± 8.1
T33	2.7 ± 0.2	5.4 ± 0.4	0.7 ± 0.1	0.03 ± 0.002	21.6 ± 1.5	70.5 ± 6.3
T100	2.7 ± 0.3	4.3 ± 0.3	0.6 ± 0.3	0.02 ± 0.004	17.1 ± 2.0	57.5 ± 5.8
T200	2.0 ± 0.3	3.7 ± 0.6	0.7 ± 0.2	0.04 ± 0.01	15.0 ± 1.7	44.7 ± 3.4

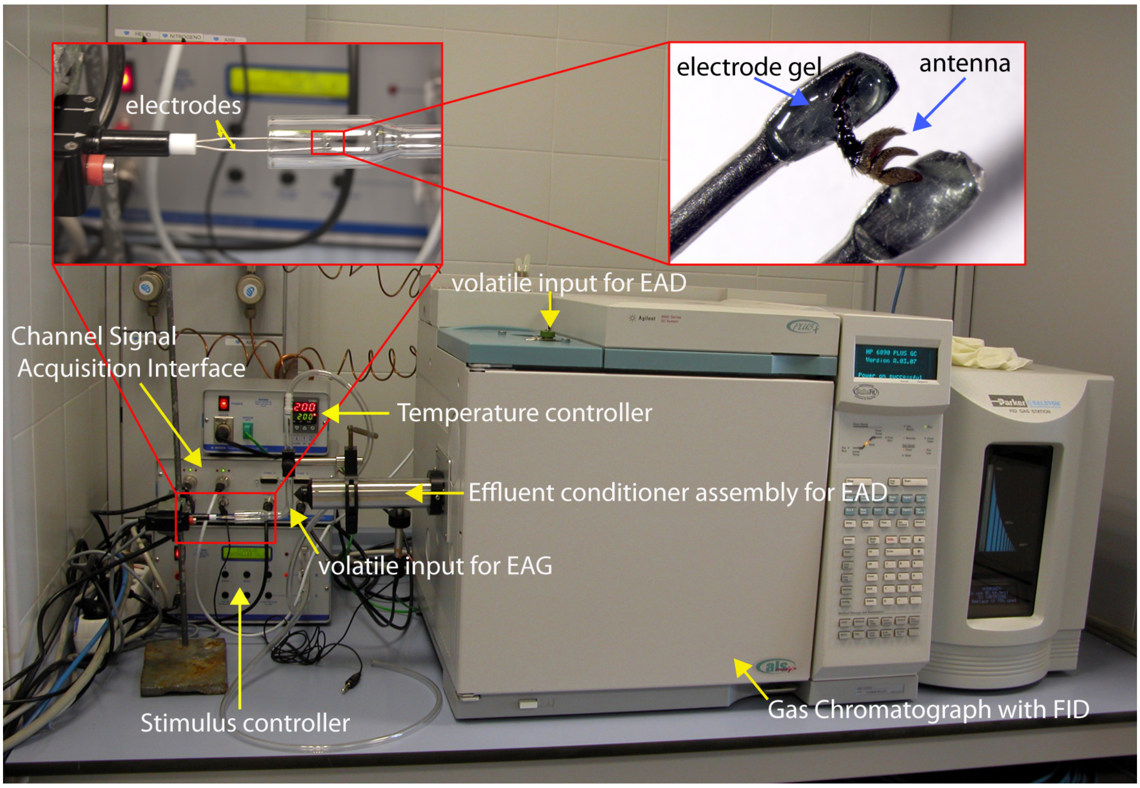

Figure S1. Experimental setup for electroantennogram recordings. Electroantennogram signals were recorded with an EAG system mounting each antenna individually between the electrodes assuring that the lamellae were well separated to optimize the reception of volatiles.

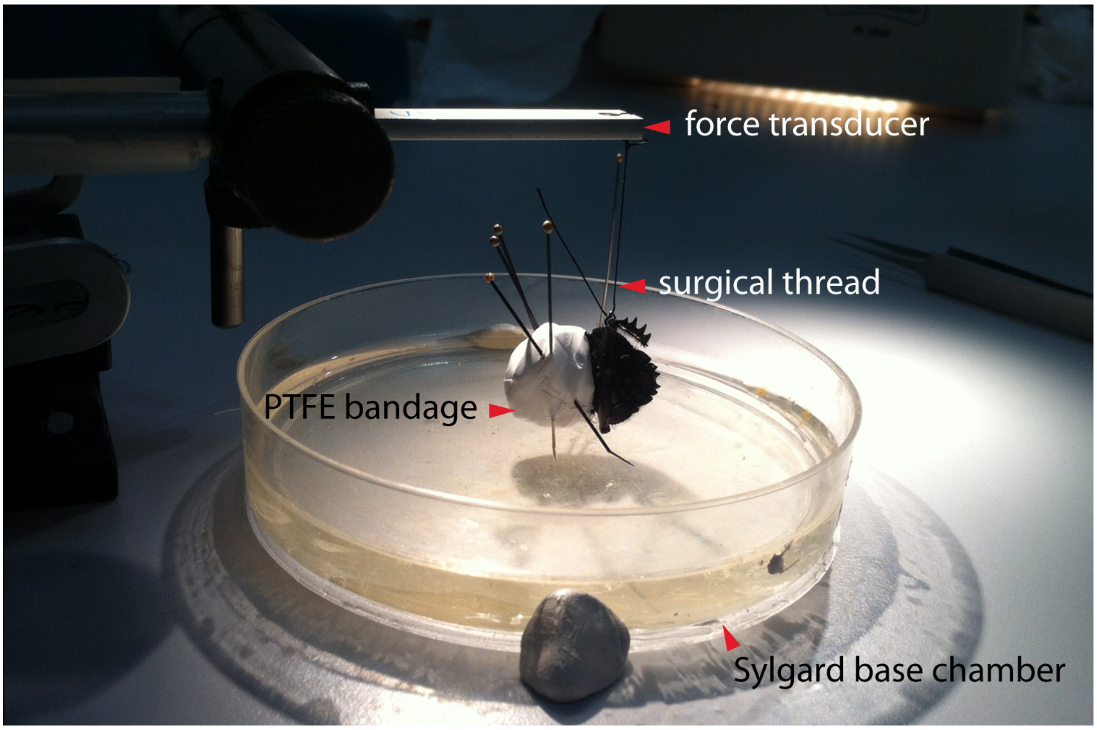

Figure S2. Experimental setup for measuring spontaneous muscle force. The beetle was immobilised using a bandage of polytetrafluoroethylene (PTFE) around the thorax and abdomen. The right foreleg of the beetle was attached to a force transducer by a surgical thread tied between the femur and the tibia articulation.

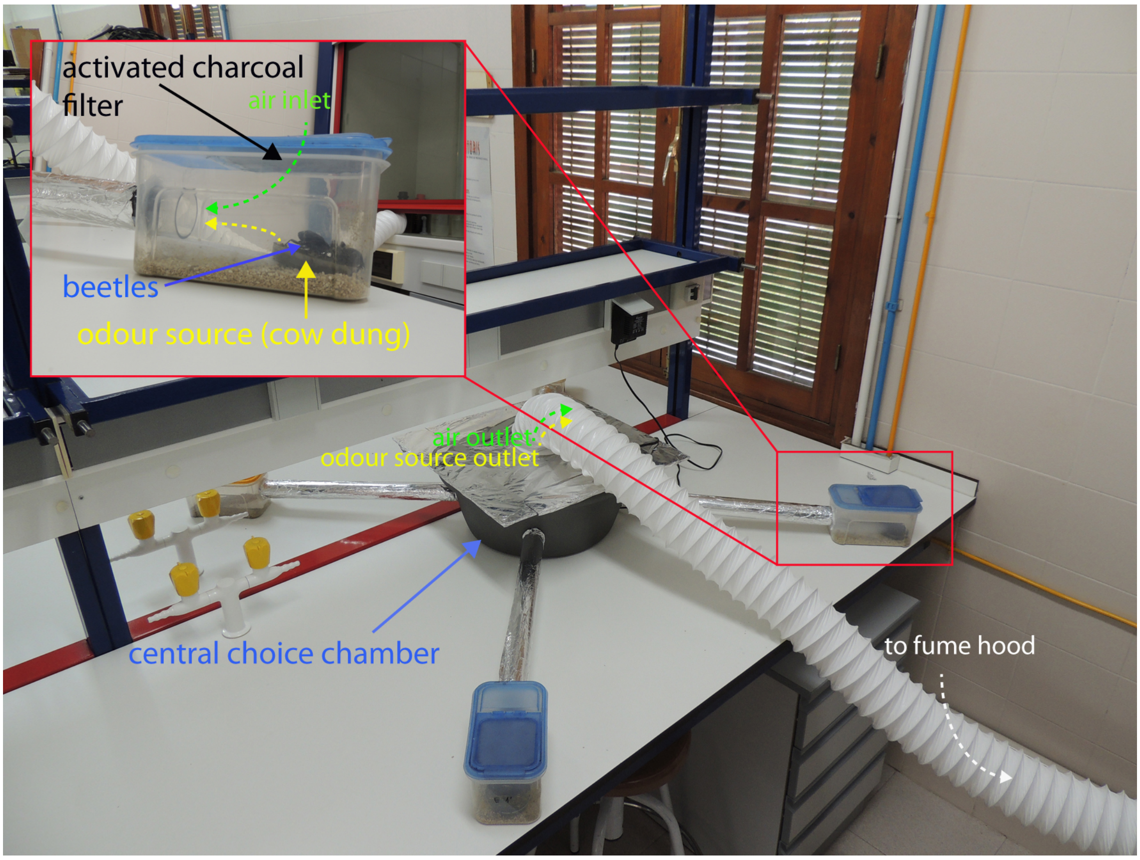

Figure S3. Experimental setup for olfactometer test. Detailed overview of the four-arm olfactometer model used in laboratory bioassays. The central arena consisted of a plastic truncate cone (60 cm superior radius and 40 cm inferior radius) with sterile vermiculite as substrate. Air, which had been passed through an activated charcoal filter, was drawn into the plastic containers of the olfactometer. In the centre of the central choice chamber there was an air out ventilator connected to a fume hood. Complete sealing of the system was ensured with the use of PTFE bandage to join all connections.
